# Supplementary material for: Services, models of care, and interventions to improve access to cancer treatment for adults who are socially disadvantaged: A scoping review protocol
Source: PLoS One. 2024 Feb 26;19(2):e0296658. doi: 10.1371/journal.pone.0296658 (PMC10896524; doi:10.1371/journal.pone.0296658)
Supplement: S1 Appendix — (DOCX) [file pone.0296658.s001.docx]

**Appendix A – Sample Search Strategy**

Ovid MEDLINE(R) and Epub Ahead of Print, In-Process, In-Data-Review & Other Non-Indexed Citations and Daily <1946 to April 04, 2023>

1 exp Neoplasms/ 3815366

2 exp Medical Oncology/ or Oncology Nursing/ 37336

3 (cancer* or neoplasm* or oncolog*).mp. 3946619

4 1 or 2 or 3 4537239

5 exp therapeutics/ or exp drug therapy/ or drug prescriptions/ or (Treatment* or therap* or chemotherap* or radiotherap* or medication* or prescription* or prescribe*).mp. 12054895

6 exp clinical trial/ or exp controlled clinical trial/ or (services or clinic* or trial*).ti. 2177684

7 5 or 6 12863004

8 exp "health disparate, minority and vulnerable populations"/ 167651

9 Medically Underserved Area/ 7469

10 alcoholics/ or exp disabled persons/ or drug users/ or exp "emigrants and immigrants"/ or homebound persons/ or exp ill-housed persons/ or refugees/ or sex workers/ or working poor/ 119067

11 "ethnic and racial minorities"/ or minority groups/ or social marginalization/ or exp socioeconomic factors/ or exp poverty/ or exp poverty areas/ or exp social class/ or low socioeconomic status/ 521462

12 Rural Population/ or exp Rural Health Services/ 80444

13 health equity/ or right to health/ 3657

14 exp health inequities/ or exp health status disparities/ or "social determinants of health"/ or healthcare disparities/ 43279

15 (social* disadvantage* or underserved or equit* or inequit*or vulnerable or homeless* or unhoused or unshelter* or hostel* or insecur* hous* or hous* insecur* or marginal* hous* or room* hous* or provisional* hous* or houseless* or emergency shelter* or provisional* accommodat* or migrant or refugee* or immigrant* or poverty or poor people or poor* neighbourhood* or poor* neighborhood* or inner city or inner cities or impoverish* or low* income or disadvantaged person* or disadvantaged people or disadvantaged neighbourhood* or disadvantaged communit* or geograph* or travel* or transportation or relocat* or racis*).mp. 631826

16 8 or 9 or 10 or 11 or 12 or 13 or 14 or 15 1295919

17 exp Home Care Services/ 50724

18 (outreach or navigat* or ambulatory).mp. 250814

19 exp Health Services Accessibility/ 134017

20 17 or 18 or 19 425373

21 4 and 7 and 16 and 20 4169

22 screening.ti. 186787

23 21 not 22 3773

24 (pediatric* or paediatric* or child*).ti. 1058049

25 23 not 24 3617

26 limit 25 to english language 3491

27 (comment or editorial or news or newspaper article).pt. 1663584

28 (letter not (letter and randomized controlled trial)).pt. 1206372

29 27 or 28 2360582

30 26 not 29 3333
