# Supplementary material for: Services, models of care, and interventions to improve access to cancer treatment for adults who are socially disadvantaged: A scoping review protocol
Source: PLoS One. 2024 Feb 26;19(2):e0296658. doi: 10.1371/journal.pone.0296658 (PMC10896524; doi:10.1371/journal.pone.0296658)
Supplement: S2 Appendix — (DOCX) [file pone.0296658.s002.docx]

**Appendix B – Preliminary Data Charting Form**

Variables for Data Charting

- Author
- Year of publication
- Type of publication
- Journal or website
- Study information: study design
- Study information:
  - Study design; conceptual/theoretical framework (if applicable)
  - Study population
  - Sample and sample size
  - Geographical location (country, region, city; urban vs. rural)
  - Timeframe of study
  - Social disadvantage – how was this determined? What forms of social disadvantage were experienced by study participants?
  - Data collection methods
- Service or model of care to improve access to cancer treatment:
  - Type of cancer treatment offered
  - Type of cancer treated
  - Description of service (narrative description; type of service or model of care used; type of staff/health care providers involved; etc)
  - Barriers addressed through service
- Evaluation of service or model of care
  - Methodology/methods used to evaluate service
  - Results of the evaluation (including strengths, limitations)
  - Key findings
  - Considerations for making changes or improvements to the service or model of care
- Additional notes
