# Supplementary material for: Services, models of care, and interventions to improve access to cancer treatment for adults who are socially disadvantaged: A scoping review protocol
Source: PLoS One. 2024 Feb 26;19(2):e0296658. doi: 10.1371/journal.pone.0296658 (PMC10896524; doi:10.1371/journal.pone.0296658)
Supplement: Attachment — Submitted filename: Response to Reviewers_PLOSOne.docx [file pone.0296658.s004.docx]

| **REVIEWER COMMENT** | **AUTHOR RESPONSE** | **LOCATION OF REVISIONS** |
| --- | --- | --- |
| **Reviewer #1** | | |
| Background: Although the concept of people who are socially disadvantaged is well-explained in the inclusion/exclusion criteria in the methodology, perhaps a definition early in the background could be provided as the concept of people who are socially disadvantaged could have different meanings in different contexts. | Thank you for this feedback. We have revised the manuscript to include the discussion of social disadvantage within the introduction:  “We understand social disadvantage as an outcome of the complex relationships between access to the social determinants of health (e.g., income, education, housing) and aspects of identity (e.g., ‘race’, gender, disability). People who are socially disadvantaged are often pushed to the margins and/or excluded from society in multiple ways, resulting in less access to resources and opportunities for health. The complex relationships between social determinants of health, social positioning, and broader social, economic, political and historical contexts are explicated in the WHO’s Commission on the Social Determinants of Health, and the Conceptual Framework for Action on the Social Determinants of Health that followed.” | Lines 54-62 |
| Objectives: the justification for conducting the scoping review is clear. However, this justification could be strengthened by indicating whether any reviews have been conducted on the topic or related topics and where gaps are. | Thank you – we have now added:  “Preliminary literature searches conducted by the academic librarian (JL) indicate there are no other planned or ongoing reviews on this topic.” | Lines 114-115 |
| Methods: although the inclusion and exclusion are well-described in terms of the concept and language, perhaps also to include what type of articles/studies/literature will be searched for. | Thank you for this suggestion. We have added an additional row to Table 1. | Line 209 |
| Limitations: limitations were clearly indicated. Perhaps also to indicate what measures/strategies will be implemented to enhance rigour of the scoping review. | Thank you for this feedback, however, it is unclear if the reviewer is requesting specific strategies to enhance the rigour of the review. If so, could you please be more specific?  At present, we have taken a number of steps to enhance rigour, including:  -Using a common methodological approach  -Describing our methodological approach in detail through this protocol  -Reporting our protocol and plans to report findings according to international standards as outlined in PRISMA extension for scoping reviews (which is currently the most up-to-date and advanced approach for scoping review reporting, and based on robust methodological guidance).  -Indicating that we will report changes/deviations from the published protocol in our report of publication of findings | N/A |
| Documentation/reporting: the how of reporting of the review was outlined, however perhaps the reporting tools (e.g., PRISMA flowchart, tables, graphs) could be included | Thank you for this suggestion, however, it is unclear what we could add at the protocol stage. We will not have a PRISMA flow chart until we have completed the database searches and 3-step screening as outlined in this protocol. Without having completed the review, we do not have any data to include in tables and graphs. | N/A |
| **Reviewer #2** | | |
| Indicate whether studies will be included or excluded based on their design and provide a rationale. For instance, will the authors include both experimental and non-experimental interventions that improve access to cancer treatment? | Thank you for this suggestion. We have now added an additional row to Table 1 outline our inclusion criteria for types of studies.  Inclusion: Peer-reviewed primary research studies (including observational studies, randomized controlled trials, quasi-experimental studies and other non-randomized trials), systematic reviews, qualitative studies and peer-reviewed quality improvement reports  Exclusion: Non-peer reviewed research, case reports, discussion papers, opinion papers, commentaries, theses/dissertations, conference proceedings, slide presentations, news stories | Line 209 |
| Study selection: Describe how duplicate records/studies will be identified and managed. | Search results will be merged and de-duplicated in Covidence software. | Line 173-174 |
| Table 1 (Inclusion and Exclusion Criteria) suggests that the review will focus on adults aged 18 years and older who are socially disadvantaged. This focus must reflect in the background, abstract and possibly the title of the review. Socially disadvantaged people may include children, adolescents or youth etc. | We have clarified our focus on socially disadvantaged adults (title, abstract, body), and included a rationale for this:  “Given that children and youth who are socially disadvantaged have entirely different experiences of disadvantage than adults, and that cancer care organizations structure adult and pediatric cancer services very differently, our focus in this review is on adults.” | Line 167-170 |
| Focus of publication: The authors indicated that “Focus is on describing a service, intervention or models of care developed and implemented with the explicit aim of improving access to, receipt of, or adherence to cancer treatment”. The concept “adherence” is introduced here for the first time in the protocol. Adherence to cancer treatment is absent in the review question, purpose or search strategy. Countless services, models of care and interventions (experimental/non-experimental) that “improve adherence to cancer treatment” exist and will considerably expand the scope and resources required of the review. Consider deleting the “adherence to cancer treatment” component. Otherwise, comprehensively integrate the “adherence to cancer treatment” component into the scoping review. | Thank you for pointing this out. We have removed reference to “adherence”. | Line 209 |
| Review the sentence “A comprehensive search strategy will be developed by an academic librarian will be developed” | Revised to: “A comprehensive search strategy will be developed by an academic librarian.” | 39 |
| Consider removing the open science registration link from the abstract | Removed. | N/A |
| Review sentence starting with “Evidence also….” | Revised to: “Evidence also suggests that race and/or ethnicity are closely linked to social disadvantage and are associated with inequities in cancer treatment.” | 69 |
| Identifying relevant studies: Indicate what action(s) will be taken if the full text of a record/study (other than grey literature and dissertations) is not found. | We have now added:  “The review team (including the medical librarian) will search for full text across all available databases and where full text is unavailable, record/study will be excluded and reported as inaccessible.” | 179-180 |
| Exclusion criteria: Provide a rationale for studies published in languages other than English language. | Studies published in languages other than English will be excluded for feasibility (limited financial and human resources). In addition, a recent meta study found that exclusion of non-English publications from systematic reviews had minimal impact (if any) on the overall conclusions drawn from the review findings (Nussbaumer-Streit et al., 2020; <https://www.sciencedirect.com/science/article/abs/pii/S0895435619306572>) | N/A |
| Consider merging the two population rows. | Revised. | 209 |
