# Supplementary material for: Services, models of care, and interventions to improve access to cancer treatment for adults who are socially disadvantaged: A scoping review protocol
Source: PLoS One. 2024 Feb 26;19(2):e0296658. doi: 10.1371/journal.pone.0296658 (PMC10896524; doi:10.1371/journal.pone.0296658)
Supplement: Attachment — Submitted filename: Response to Reviewers R2_PLOSOne.docx [file pone.0296658.s005.docx]

| **REVIEWER COMMENT** | **AUTHOR RESPONSE** | **LOCATION OF REVISIONS** |
| --- | --- | --- |
| **Reviewer #3** | | |
| I would just suggest to consider anchoring the definition of the population “socially disadvantaged” which is well done in the methods and population definition in the body of the paper to be done early on in the manuscript in the abstract as well as the introduction so that the reader has a very clear understanding of how your population is defined.” | Thank you for this suggestion. We recognize this was missing from the abstract, and have revised it to offer a brief explanation of social disadvantage, which is more fully discussed in the body of the paper:  “Among people experiencing the intersecting impacts of poor access to the social determinants of health and personal identities typically marginalized from society (‘social disadvantage’), there are significant barriers to accessing cancer…”  Our introduction already includes a discussion of how we are conceptualizing social disadvantage in this protocol, which fairly closely matches our definition outlined in the methods:  “We understand social disadvantage as an outcome of the complex relationships between access to the social determinants of health (e.g., income, education, housing) and aspects of identity (e.g., ‘race’, gender, disability). People who are socially disadvantaged are often pushed to the margins and/or excluded from society in multiple ways, resulting in less access to resources and opportunities for health [2]. The complex relationships between social determinants of health, social positioning, and broader social, economic, political and historical contexts are explicated in the WHO’s Commission on the Social Determinants of Health [3], and the Conceptual Framework for Action on the Social Determinants of Health that followed [4].” (Lines 53-61)  We also explicitly apply this conceptualization to a brief discussion of inequities in cancer treatment, highlighting how poverty, housing, and race all impact access to cancer treatment, and cancer outcomes (Lines 63-82).  Some minor revisions have been made throughout the first paragraph to more explicitly link this conceptualization/definition to what is offered in the Methods section. | Lines 27-29  Lines 63-104 |
| “Another suggestion would be to provide a bit more context to geography and region – we see that the literature will be searched for English language having said that is there an assumption that the context is North America or international in terms of how we define populations globally with respect to socially disadvantaged and cancer screening as the multiple components of how you define social disadvantage cannot be separated from national/geographical and political contexts that may vary globally.” | Thank you; we agree that clarity is lacking in this area. We have clarified that our context is not limited to North America, and we are interested in learning from geographically diverse contexts:  “Although our programs of research are primarily located within the Western context (North America and Europe), we are interested in learning from and about services and models of care within and beyond this context (globally).”  “In this review, we are interested in people who are marginalized by way of one or more forms of social disadvantage, *recognizing that how social disadvantage manifests may differ depending on context, but crosses geographical borders, and is not limited to a particular nation or region*.” | Lines 113-115  Lines 168-169 |
| “The appendices are referred differently in the body – i.e. Appendix A in the body is actually Appendix B”. | Thank you for highlighting this discrepancy, which we have now addressed. | Line 207 |
